# SensorAI: A Machine Learning Framework for Sensor Data

**DOI:** 10.3390/s25196223

**Published:** 2025-10-08

**Authors:** Stephen Coshatt, He Yang, Shushan Wu, Jin Ye, Ping Ma, Wenzhan Song

**Affiliations:** The Center for Cyber-Physcial Systems, University of Georgia, Athens, GA 30602, USA; stephen.coshatt@uga.edu (S.C.); heyang95@uga.edu (H.Y.); shushan.wu@uga.edu (S.W.); jin.ye@uga.edu (J.Y.); pingma@uga.edu (P.M.)

**Keywords:** artificial intelligence, digital signal processing, false data injection attack, machine learning, sensor data, time series

## Abstract

As machine learning and artificial intelligence are being integrated into cyber-physical systems, it is becoming important for engineers to know and understand these topics. In particular, sensor data is on the rise in these systems and therefore engineers need to understand which models are appropriate to time-series sensor data and how signal processing can be used with them. The Center for Cyber-Physical Systems (CCPS) at the University of Georgia (UGA) is addressing these issues. Student researchers in the CCPS require skills in these areas. This paper demonstrates a machine learning framework for time-series sensor data that can be used to quickly build, train, and test multiple models on CCPS testbed data. The framework is also a tool that can be used as a tutorial to help student researchers understand the concepts required to be successful in the CCPS.

## 1. Introduction

Artificial intelligence and machine learning are becoming ubiquitous in modern industries [1], often referred to as Industry 4.0 [2,3,4]. In particular, this includes the use of sensors in many fields, such as the Internet of Things, manufacturing, power, medical systems, digital twins, and other smart systems. Sensor data is rapidly becoming the most common form of big data [5].

Industry has seen an increasing number of electronic control units (ECUs) and programmable logic controllers (PLCs), and other types of programmable electronics have been deployed into cyber-physical systems. While such progress increased productivity and product quality, it also introduced vulnerabilities to both hardware and software.

In response to this issue, the Center for Cyber-Physical Systems (CCPS) is developing a testbed containing motors in order to collect realistic data for analysis and development of artificial intelligence and machine learning algorithms to solve fault and attack detection and diagnosis problems. The CCPS has also developed a smart plug, called ElectricDot, that can be used to easily integrate new devices into the testbed. Any device that uses a standard wall outlet may plug into an ElectricDot and be monitored by the testbed.

CCPS research requires skills in Machine Learning, Deep Learning, and Digital Signal Processing (DSP). Generally, CCPS students are electrical or computer engineers, computer scientists, and occasionally statisticians. Most students have some background in signal processing or some background in machine or deep learning. Few have expertise in both. Thus, the CCPS students must learn these topics, generally via classes, while performing research. However, these topics are generally taught in classes that are independent of each other, and their interrelation is not formally discussed. Thus, their interrelations have been discussed during lab hours or are conducted as an independent study. Therefore, the CCPS has started to formalize training and education of the topics.

The CCPS primarily deals with sensor data gathered from cyber-physical systems and uses it with artificial intelligence and machine learning to perform research. While there are a multitude of definitions for cyber-physical systems (CPS) [6], the Sensorweb Lab defines them as any networked system that affects the physical world. Thus, the lab also primarily works with streaming data. Streaming data is a unique type of time-series data in that it is continuously generated, can only be examined in one pass, and is prone to concept drift [7].

A need has arisen for CCPS researchers to have a framework that allows easy integration of data collection, algorithm training, and testing. Additionally, the CCPS has developed smart plugs, called ElectricDot (eDot), to easily connect to electronic devices for monitoring their power electric waveform data. Thus, this phase also includes adding the capability to easily connect models to eDots and other sensor devices. This paper presents a solution to these issues.

## 2. Materials and Methods

### 2.1. Background

Development of the CCPS testbed has proceeded in phases. Initially, a small prototype test bed involving toy motors was built as a proof of concept and is detailed in [8]. In this phase, a small information technology (IT) and operational technology (OT) network was created, with the motors at the OT level. A multilevel cyber attack was created. In short, an attacker was able to access the network via WiFi, scan the IT network for hosts, use a brute force password attack to break into Raspberry Pis with weak passwords, and load an attack script. The script could then send commands to the motors on the OT network.

For defense, there were anomaly detection algorithms monitoring the network traffic, the system statistics of the Raspberry Pis, and the power usage of the motors via sensors. The goal was to demonstrate a multilevel attack on an OT/IT network and demonstrate multilevel detection.

The next phase involved moving from toy motors to industrial motors in a testbed. Details of this phase can be found in [9]. In this phase, the same basic attacks were implemented at the network level. However, a more sophisticated attack was developed for manipulating the industrial motors. Care was taken to ensure the attacks did not damage the motors. Compromised firmware and a control script, which was a compromised version of open-source software for the control system vendor, were created in lieu of the basic attacks for the toy motors.

In both of these phases, live data was streamed to InfluxDBm, and Grafana was used for visualization. However, at these phases, data for training anomaly detection algorithms were simulated offline. Models were developed, trained, and tested offline as well.

This paper discusses the current phase of the CCPS testbed, the development of a framework. The framework is intended to help CCPS student researchers learn and understand relevant concepts to aid them in quickly training and testing models for more comparison in CCPS research. The development of the framework was divided into two parts: the creation of its core functionalities along with a tutorial, and the addition of the GUI with testbed integration via ElectricDots.

### 2.2. Previous Efforts with Tutorials

The Sensorweb Laboratory has made previous efforts to create tutorials to aid student researchers. These initially began assignments were that lab members would create a small tutorial on a specific model. These were presented in lab meetings. They were later compiled and placed on a small website. However, tutorials were not standardized in format. Additionally, the code from one tutorial was not easily usable by another. Thus, it was decided to consolidate the code into the framework and standardize the tutorial information into it as well.

### 2.3. CCPS Testbed Data Collection

The CCPS uses Message Queuing Telemetry Transport (MQTT) to send data and commands between sensors and systems. MQTT uses a publish/subscribe approach to handling messaging. Devices and apps subscribe to a topic to receive messages and publish to a topic to send. All messages and subscriptions are handled by an MQTT broker. InfluxDB, a free time-series database, is used to store data. Grafana, a free tool, is used for data visualization of live streaming devices. Applications and smart devices used by the CCPS send data to the MQTT broker, which in turn forwards data to the InfluxDB servers. The testbed is located in a lab on the University of Georgia’s (UGA) campus and is connected to the university’s secure network. The Grafana and InfluxDB servers reside in this network and are not accessible from outside the network.

### 2.4. ElectricDot

**Figure 1:**
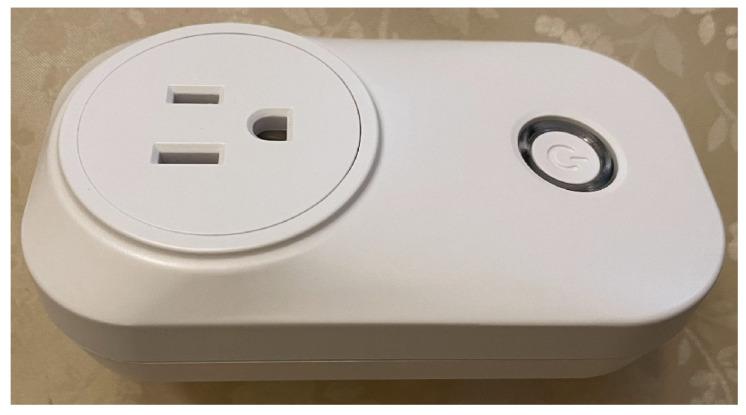
Picture of an ElectricDot smart plug.

ElectricDot is a smart device designed to monitor the power electric waveform data of devices plugged into it. An image of an ElectricDot is shown in Figure 1. ElectricDot is plugged into a wall socket, and the device that one intends to monitor is then plugged into the ElectricDot. Data is sent over WiFi via MQTT messages. The ElectricDot can be programmed to a sample rate up to 10 kHZ. Additionally, various types of feature extraction, such as amplitude, frequency, phase, and angle, can be programmed as well. The ElectricDot was designed by CCPS in a separate project. Its purpose is to easily provide anomaly detection and diagnosis for electric devices and networks. They allow for CCPS researchers to quickly add a power sensor to any device, including devices within the CCPS testbed. Thus, the SensorAI Framework is required to have the capability to interface with them.

### 2.5. Design Requirements

The projects and research discussed in this paper are the efforts of the Sensorweb Laboratory and the Intelligent Power Electronics and Electric Machine Laboratory. Both of which are members of the CCPS. In discussions with lab leaders and members, the following high-level requirements were determined for the framework:

- Must use Python.
- Must focus on time-series/sensor data.
- Minimize coding.
- Easy to use.
- Must assist in training and testing.
- Must include metrics.
- Visualization of data and results.
- Must have an accompanying tutorial.
- Must include digital signal processing, classification, clustering, regression, and anomaly detection.
- Must have a Graphical User Interface.
- Core functionality must be directly accessible.
- Must enable smart device and historical data connectivity to models.

### 2.6. Comparable Frameworks

There are many frameworks and automated machine learning software available. To narrow down a review of available products, the requirements listed above were used. In addition, only free open-source products were considered, due to limited funds and to avoid extra costs for student researchers. Thus, products such as Microsoft Azure ML Studio [10] were not considered. Products not written in Python, such as WEKA Workbench [11], were not considered. H20 AutoML [12], TPOT [13], AutoGluon [14], and Auto-sklearn [15] are three comparable products that meet some of the requirements above. TPOT, AutoGluon, and Auto-sklearn do not include digital signal processing capabilities. H20 AutoML has DSP capabilities; however, the free version does not. Thus, the CCPS was motivated to create its own framework.

### 2.7. Design Part 1: Core Functionality and Tutorial

The framework is built with Python. The primary interface for the framework is conducted via a browser to a Streamlit web application server. Streamlit is an open-source Python library for creating web applications. Note that the underlying code for the framework can be executed directly on a workstation with an Integrated Development Environment (IDE) or via a Google Colab ^TM^ notebook. It primarily uses the SciKit Learn [16] and SciPy [17] packages, although others are used. Most models incorporated into the framework are SciKit Learn. Any model with Time Series in its name is from tslearn [18], which is an extension of SciKit Learn designed specifically for time-series data. Users may upload their own data or generate data within the framework. Visualizations are conducted with matplotlib [19] and Plotly v6.3.0 [20].

A complete list is contained in the *requirement.txt* file on the SensorAI GitHub, version 9/29/2025 [21]. The code is divided into its core tasks. Digital Signal Processing, Classification, Clustering, Regression, Anomaly Detection, and Utilities; refer to Table 1. Each machine learning module contains code for the easy creation of pipelines and grid searches so that researchers can quickly train, test, and compare multiple models with minimal coding. Utilities contains the visualization and other miscellaneous functions that the core modules need. In general, a user will not need to access Utilities directly, but may do so if they choose.

**Figure 2:**
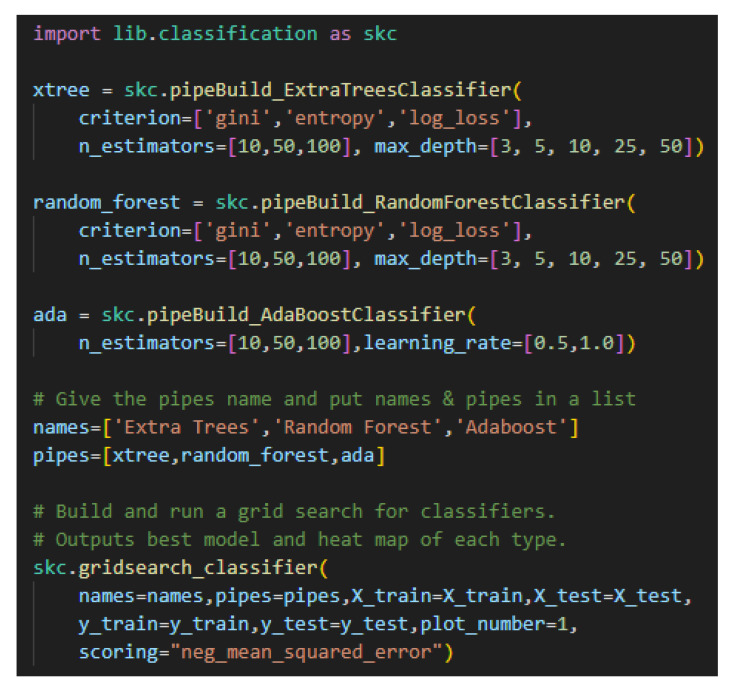
An example of framework code. Extra Trees, Random Forest, and AdaBoost decision tree classifiers are created and then placed in a grid search. Note that hyperparameter values for the grid search are placed in lists.

For the core modules, there is very little coding required to build a model pipeline and place it in a grid search. Figure 2 shows the code for setting up a grid search for three models: Extra Trees, Random Forest, and an AdaBoost decision tree.

The rationale for this organization was based on the CCPS’s need to ensure that student researchers understand the core concepts. It also made integration of various algorithms easier, as models of each category generally rely on the same underlying utilities, such as metrics and visualization.

Most functions in DSP have a Show option, which is set to True or False, that allows the user to decide whether or not to plot the results of the operation. Thus, users only need to set a variable plot instead of having to write multiple lines of code to perform the same plotting.

#### 2.7.1. Digital Signal Processing

The DSP module contains functions for all of the signal processing used in the framework. The groupings discussed in this section follow the general outline of the associated tutorial.

Wave generation: Wave generation allows users to create waves, such as sine, square, triangle, as well as pulses and chirps. Various types of noise can be added to these waves. Noise types include, but are not limited to, white, flicker, impulse, and echo. There are also functions that allow users to automatically generate sets of waveforms, with or without noise, to create synthetic datasets that may be used for model training and testing, as well as with tutorials. It also provides functions to generate the seismocardiography (SCG) signal of a heartbeat, with or without respiration.

Filters and Signal Averaging: The module includes basic linear filters such as high-pass, low-pass, band-pass, and band-stop. It also includes more complex filters such as adaptive, moving average, and Kalman. Signal averaging contains several methods of dynamic-time-warping averaging techniques.

Time and Frequency Domains: The DSP module includes statistical moments, peak detection, envelope extraction, and waveform complexity measures. It also contains the Fast-Fourier Transform (FFT), the short-time Fourier transform (STFT), and power spectral density functions. There are also functions to extract the average amplitude, frequency, and phase of a waveform, as well as a function to calculate the Total Harmonic Distortion (THD) of a waveform.

Signal Decomposition: Within the module, there are functions to allow the user to break down (decompose) a signal into its fundamental parts and plot them. It includes Empirical Mode Decomposition (EMD), EMD variations, Singular Spectrum Analysis (SSA), and various Blind Source Separation techniques.

Wavelet Analysis and Transforms: Multiple types of wavelets and chirplets are available in the module. Additionally, the following wavelet-based transforms are in this module: Continuous Wavelet Transform (CWT), Polynomial Chirplet Transform (PCT), Wigner–Ville Distribution (WVD), and SynchroSqueezing Transform (SST).

#### 2.7.2. Classification

This module contains all the classification models available in the framework. It also contains the functions for streamlining pipeline building and grid searches. The groupings discussed in this section follow the basic outline of the associated tutorial. These models can also be used for anomaly detection (see Section 2.7.4 below).

Decision Trees, Bagging, and Boosting: In addition to basic decision trees, the framework includes several tree-based bagging methods: Random Forest and Extra Trees. It includes two tree-specific boosting methods: Gradient Boosting and Histogram Gradient Boosting. It includes a generic bagging model that can create ensembles of other model types called bagging. Lastly, it includes a generic boosting model that can boost other model types called AdaBoost.

Nearest Neighbors: This module contains several nearest neighbor-based models. They are K Nearest Neighbors (KNNs), Nearest Centroid, Radius Nearest Neighbors, and Time-Series K Nearest Neighbors (TS KNNs).

Support Vector Classifiers: The framework contains three support vector-based models: Support Vector, Nu Support Vector, and Time-Series Support Vector.

Other Classifiers: There are multiple other classifier models available, which include but are not limited to Discriminant Analysis, Early Classifiers, and Naive Bayes classifiers.

#### 2.7.3. Clustering

This module contains all the clustering models available in the framework. It also contains the functions for streamlining pipeline building and grid searches. The groupings discussed in this section follow the basic outline of the associated tutorial.

Hierarchical Clustering: In the tutorial, Hierarchical Clustering is used as an introduction to clustering, as these models are simple. Agglomeration and Feature Agglomeration are available in the framework.

K-Means: K-Means is another easy clustering model to understand. The framework includes the following K-Means-based variants: K-Means, Bisecting K-Means, Mini-Batch K-Means, Time-Series K-Means, and K-Shape.

Density-Based Clustering: The density-based clustering algorithms available in the framework are Mean Shift, Density-Based Spatial Clustering of Applications with Noise (DBSCAN), and Ordering Points To Identify the Clustering Structure (OPTICS)

Spectral Clustering: Spectral Clustering is the only other clustering algorithm currently available in the framework.

#### 2.7.4. Detection

Unsupervised anomaly detection is divided into Outlier Detection and Novelty Detection. In outlier detection, the goal is to identify anomalous data within your dataset, usually with the intent to remove them. In novelty detection, the goal is to build a model that can label future data inputs as either normal or anomalous.

In this framework, supervised anomaly detection is part of classification. Any classification method can be an anomaly detection method if the normal condition is one class and all other conditions are treated as anomalous.

Outlier Detection: Unsupervised outlier detection is contained in the Detection module. The detectors are Local Outlier Factor, and Elliptic Envelope.

Novelty Detection: Unsupervised novelty detection is contained in the Detection module. The detectors are One-Class Support Vector Machine, One-Class Support Vector Machine with Stochastic Gradient Descent, Isolation Forests, and Local Outlier Factor for Novelty Detection.

#### 2.7.5. Regression

This module contains all the regression models available in the framework. It also contains the functions for streamlining pipeline building and grid searches. The groupings discussed in this section follow the basic outline of the associated tutorial. When using the tutorial, regression is generally used first since it contains discussions on probability, distributions, loss functions, cross-validation, and regularization.

General Linear Models: The general linearized models available in the framework are Linear, Gamma, Poisson, and Tweedie.

LARS and LASSO: Least Angle Regression Shrinkage (LARS) and Least Absolute Shrinkage and Selection Operator (LASSO) are included together. There are several variants of the models included in the framework: LARS, LARS with Cross Validation, LASSO, LASSO with Cross Validation, LassoLars, and LassoLars with Cross Validation.

Ridge: The Ridge-based models available are Ridge, Ridge with Cross Validation, and Bayesian Ridge.

Elastic Net Regularization: Elastic Net Regularization (Elastic-Net) is a combination of LARS and Ridge. There are several variants of Elastic-Net available: Elastic-Net, Elastic-Net with Cross Validation, Multitask Elastic-Net, and Multitask Elastic-Net with Cross Validation.

Support Vector Regression: The support vector regressor models included are Linear Support Vector, Nu Support Vector, and Time-Series Support Vector.

Other Regression Methods: The list of other regressors available includes, but is not limited to, Huber, TheilSen, Random Sample Consensus (RANSAC), Time-Series KNN regressor, and Quantile.

#### 2.7.6. Utilities

This module mostly contains the various plotting functions and some metric calculations. It has a few miscellaneous functions that do not fall into any of the other categories in the framework. The plotting functions primarily used are for confusion matrices and waveform data display.

#### 2.7.7. Framework Tutorial

Each of the core machine learning modules of the framework has a companion tutorial on how to use it; refer to Table 2. Clustering and Unsupervised Anomaly Detection are combined into the Unsupervised tutorials. Supervised Anomaly Detection is covered under the Classification tutorials. The tutorial includes overviews of the concepts and algorithms of machine learning. There is a set of slides and a Jupyter notebook with executable framework code for each module.

The general outline of each tutorial follows the topics grouped in the discussion of each module in the code design section. The outline below highlights the main topics on which each tutorial is focused.

- DSP: wave generation, noise, filters, transforms, decomposition, power spectral density, wavelet analysis, and transforms.
- Classification: decision trees, nearest neighbors, support vector machines, bagging, boosting, others.
- Clustering: hierarchical, k-means, k-shape, density-based, spectral, others.
- Anomaly Detection: outlier vs. novelty detection, isolation forests, local outlier factor, others.
- Regression: generalized linear models, lars, lasso, ridge, elastic nets, nearest neighbors, support vector machines, others.

### 2.8. Design Part 2: Graphical User Interface and Testbed Integration

**Figure 3:**
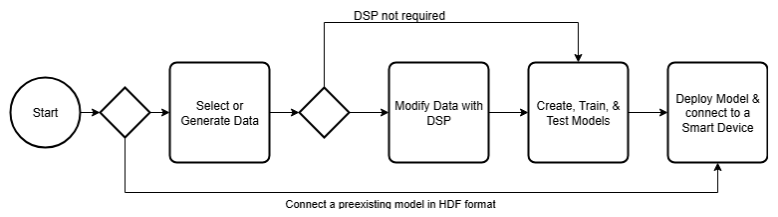
The overall flow fo the framework using the GUI.

A free Python package, Streamlit, was chosen for the GUI. Streamlit is for building graphical interfaces to Python code that are accessed via a web browser. It was chosen for ease of use and minimization of coding. Figure 3 illustrates the overall flow of the framework.

**Figure 4:**
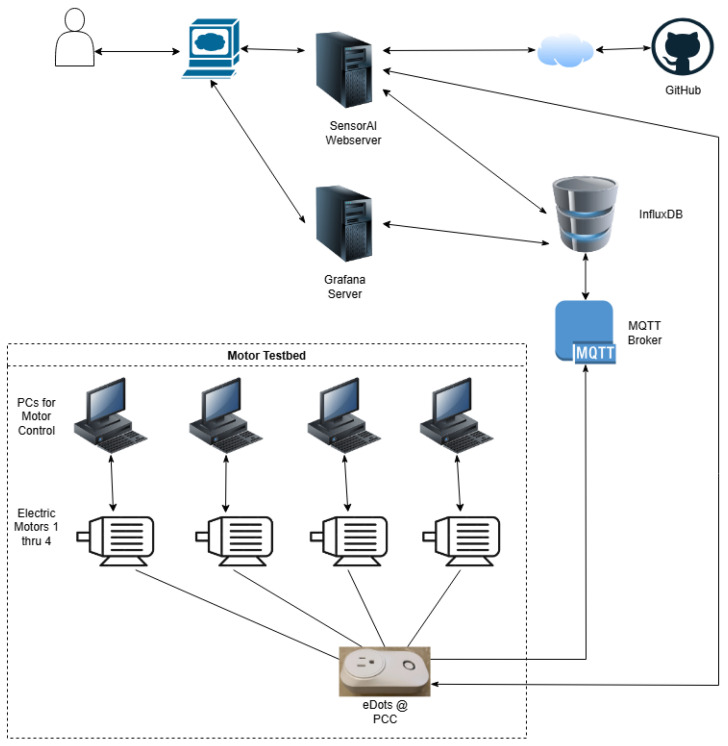
SensorAI Framework and testbed integration: The SensorAI Framework is hosted on a server that resides in UGA’s secure network. All PCs, servers, and brokers are also hosted on UGA’s secure network. The motors, the PCs used for motor control, and the ElectricDot are all physically located in the same lab on campus. Note that the ElectricDots smart plug is connected to the PCC that all four motors are connected to.

The graphical user interface for the framework follows the basic architecture of the core framework code. There are pages for digital signal processing, classification, clustering, detection, and regression. Additionally, there are pages for loading and generating data, downloading data from InfluxDB and running it through an existing model, and one for connecting an active ElectricDots to a trained model and sending model outputs to the MQTT broker for forwarding to the appropriate InfluxDB server. It also allows for the connection of a model to historical data from the InfluxDB server. Lastly, there is a page for the tutorials. The SensorAI Framework is integrated with the motor testbed, as shown in Figure 4 below.

**Figure 5:**
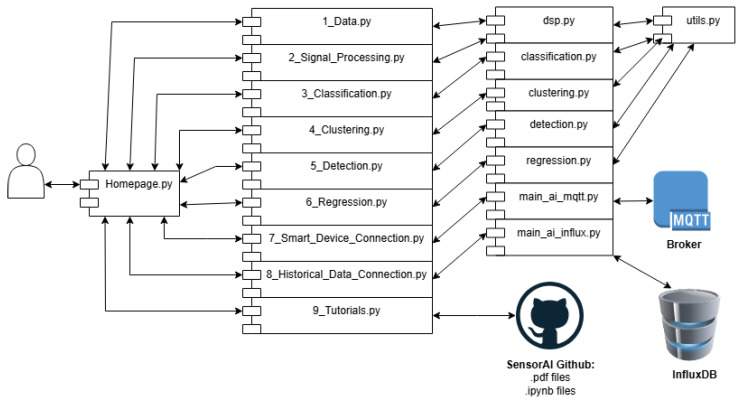
Framework Code Design: Homepage.py is the main Streamlit page. The functionality is divided into nine subpages. Each subpage is titled with a number followed by an underscore. Streamlit uses this numbering for page ordering. Each page is a graphical interface to the underlying functional code or to the tutorials.

The framework GUI may be run via the Homepage.py file. This file sets the basic layout and color schemes. Each page, except for the Device Connector and Historical Data Connector, has an associated Python file that contains most of the related GUI functionality. This is conducted to avoid overly large files for ease of maintenance and understanding. The code design for the framework is illustrated in Figure 5. Within the testbed, the framework is hosted on a server running the Streamlit code.

#### 2.8.1. Data

**Figure 6:**
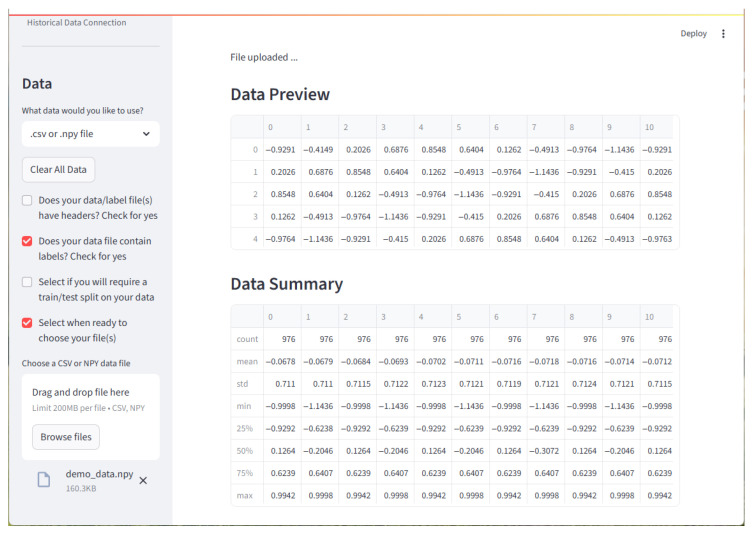
The image is of the Data page after a demo dataset has been loaded. Note that options are selected in the left sidebar. Once data is selected, a preview of the first five entries is displayed. A data summary is displayed below it. While not shown in the screenshot, the first row of data is also plotted.

The Data page is the second in the framework. Here, users can choose to load data from numpy data (.npy) files or a comma-separated values (.csv) files. Alternatively, they may generate a single waveform or a set of multiple waveforms. Lastly, users may generate seismocardiography data. Either a single example or a dataset of randomly generated ones. The Data page can be seen in Figure 6.

#### 2.8.2. Digital Signal Processing

**Figure 7:**
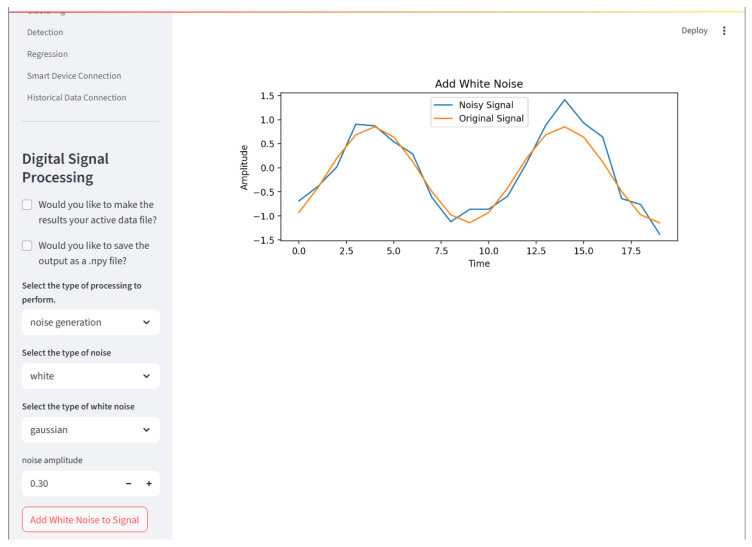
The Signal Processing page is where digital signal processing techniques may be applied to a loaded dataset. Here, basic Gaussian white noise is added to the demonstration dataset. Note that the first entry of the dataset is plotted. Both the original signal and the signal with added noise are shown.

The *DSP* page contains all the functions for digital signal processing. It is segregated by sub menus: Noise, Filters, Decomposition, Time Domain Features, Transforms, and Misc. The DSP menu can be seen in Figure 7.

#### 2.8.3. Classification

**Figure 8:**
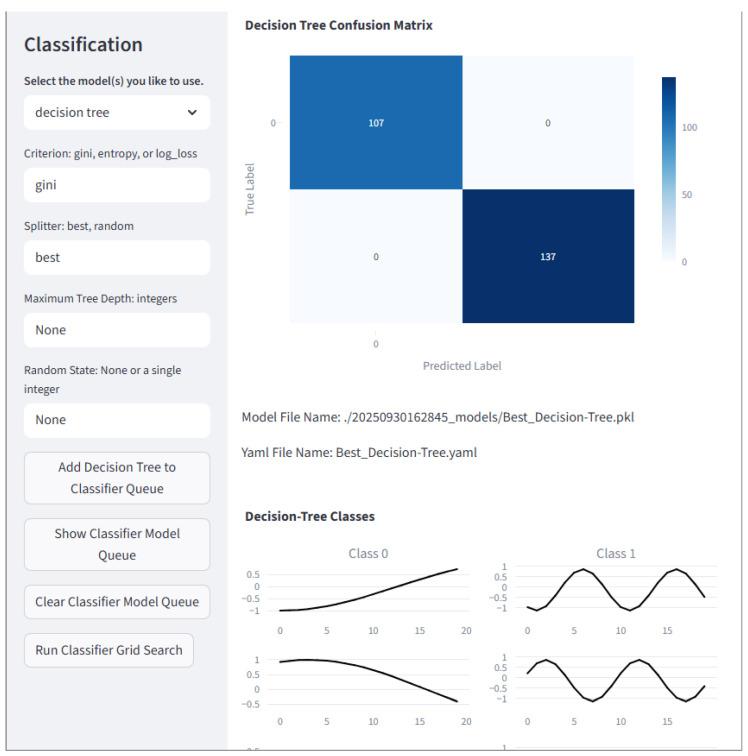
The Classification page is where classification algorithms may be created and queued to run on a loaded dataset. Mul tiple inputs for each hyperparameter of multiple models may be created and run through a grid. The best model of each type placed in the queue will have results displayed. Here, a confusion matrix and the first three samples of each class are displayed. Note that plots in black are correctly classified, and those in red are incorrectly classified.

The Classification page offers several models that users may train and test. They may use their own data or data generated by the framework. Related models are grouped in columns. The Classification page is shown in Figure 8. Note that any classification model may be used as novelty detection in the case of having training data with normal and abnormal labels.

#### 2.8.4. Clustering

**Figure 9:**
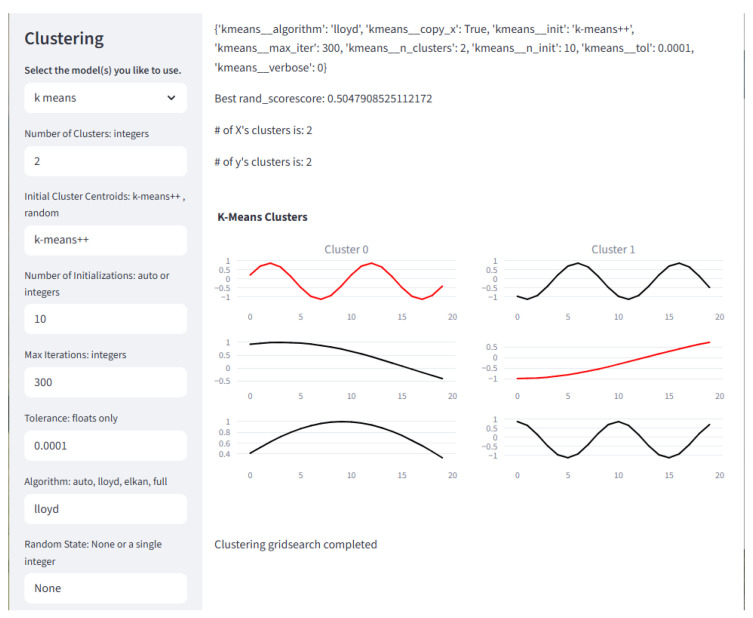
The Classification page is where clustering algorithms may be created and queued to run on a loaded dataset. Multiple inputs for each hyperparameter of multiple models may be created and run through a grid search. The best model of each type placed in the queue will have results displayed. Here, the first three samples of each cluster are displayed. Note that plots in black are correctly clustered, and those in red are incorrectly clustered.

The Clustering page offers several models that users may train and test. They may use their own data or data generated by the framework. Related models are grouped in columns. The Clustering page is shown in Figure 9.

#### 2.8.5. Detection

**Figure 10:**
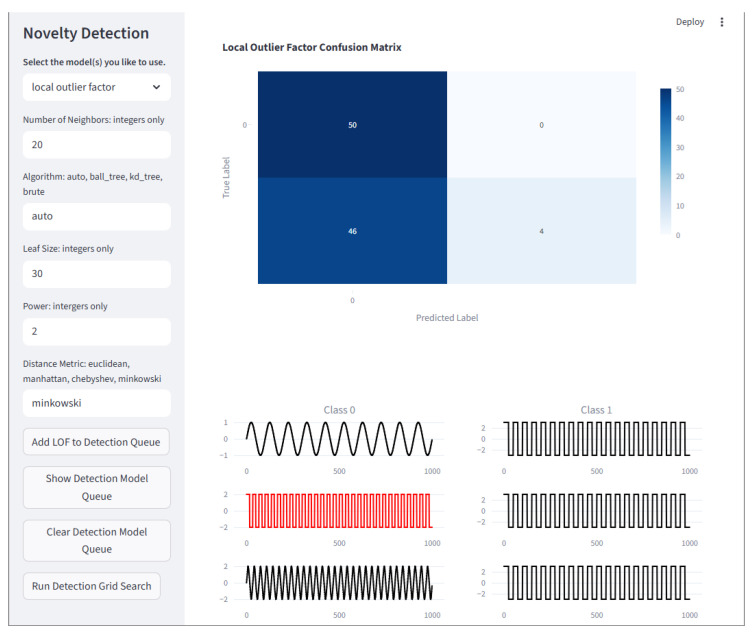
The Detection page is where outlier and novelty detection algorithms may be created and queued to run on a loaded dataset. Multiple inputs for each hyperparameter of multiple models may be created and run through a grid search. The best model of each type placed in the queue will have results displayed. Here, a confusion matrix and the first three samples of each class are displayed, where the first column is normal (sine waves) and the second column is abnormal (square waves). Note that plots in black are correctly identified, and those in red are incorrectly identified.

The Detection page has two sub-menus, Novelty and Outlier. These menus contain a few models that are specific to these tasks. The Detection page is shown in Figure 10.

#### 2.8.6. Regression

**Figure 11:**
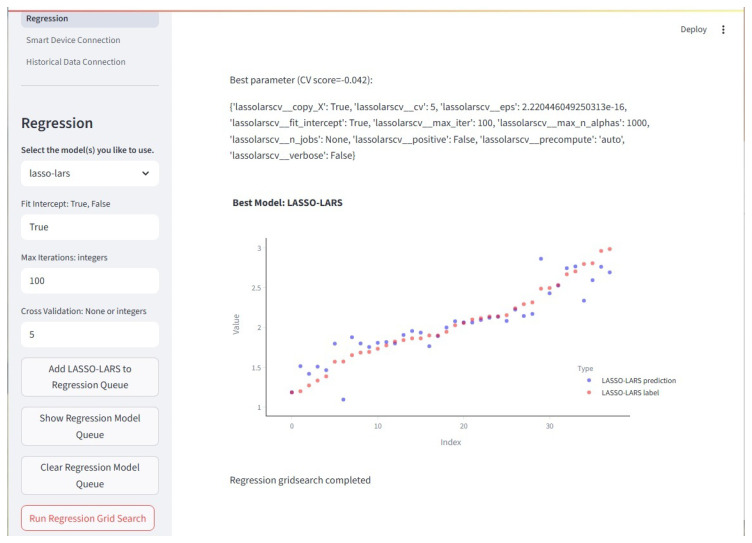
The Regression page is where regression algorithms may be created and queued to run on a loaded dataset. Multiple inputs for each hyperparameter of multiple models may be created and run through a grid search. The best model of each type placed in the queue will have results displayed. Here, a scatterplot is displayed. Note that predicted values are blue and the labels (true values) are red.

The Regression page offers several models that users may train and test. They may use their own data or data generated by the framework. Related models are grouped in columns. The Regression page is shown in Figure 11.

#### 2.8.7. Device Connector

**Figure 12:**
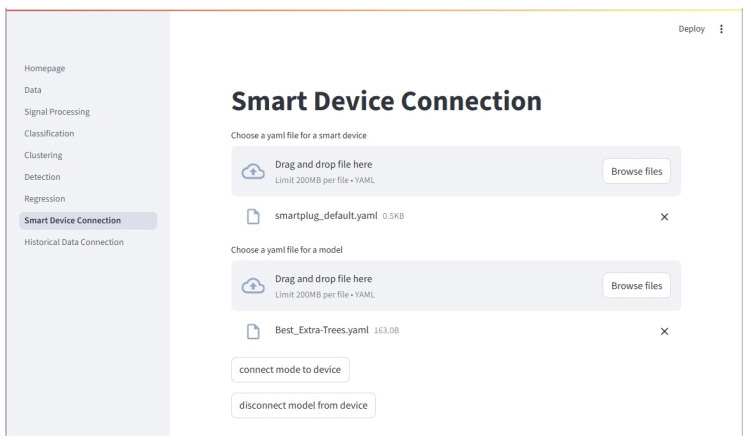
The Device Connector page is where a trained model may be connected to a live ElectricDot (or other smart device) data stream. The model and the smart device each have an associate .yaml file that contains the information that the framework needs to make the connection. Based on the information in the .yaml file, the model output is automatically routed to the appropriate InfluxDB server and table. From there, Grafana may be used to visualize the smart device and model outputs in real time.

The Device Connector page allows the user to connect a trained model to an online ElectricDots. Models trained and saved by the framework are pickled, and an accompanying yaml file with model information is generated. The user may connect any of their own models, so long as it is in pickled format and the user has created an appropriately structured accompanying yaml file. The page is shown in Figure 12. When connecting a model to an ElectricDot, a separate process is opened and the main_ai_mqtt.py script is automatically run with the appropriate settings. A separate terminal window opens and displays any text output. The connection can be monitored from this terminal window.

#### 2.8.8. Historical Download

**Figure 13:**
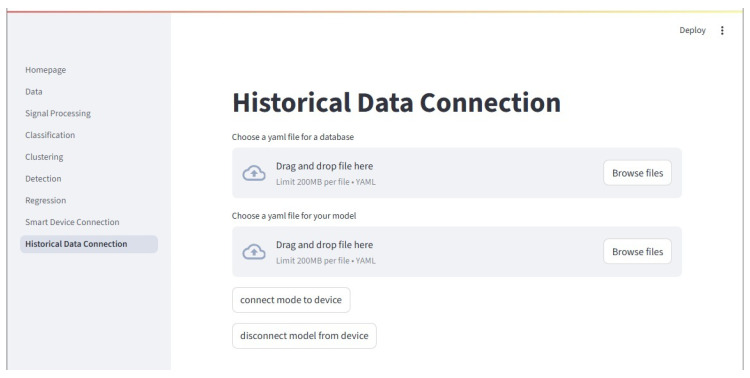
The Historical Data page is where a trained model may be connected to historical data stored in the testbed’s InfluxDB server. The historical data is queried and sent to the model. It otherwise functions the same as the Device Connector discussed above.

The Historical page allows users to download and pass historical data from the InfluxDB server to a model of their choosing. As with the previous section, the models must be pickled and have their accompanying yaml file. This page is shown in Figure 13. When connecting a model to historical InfluxDB data, a separate process is opened and the main_ai_influx.py script is automatically run with the appropriate settings. A separate terminal window opens and displays any text output. The connection can be monitored from this terminal window.

#### 2.8.9. Tutorials

**Figure 14:**
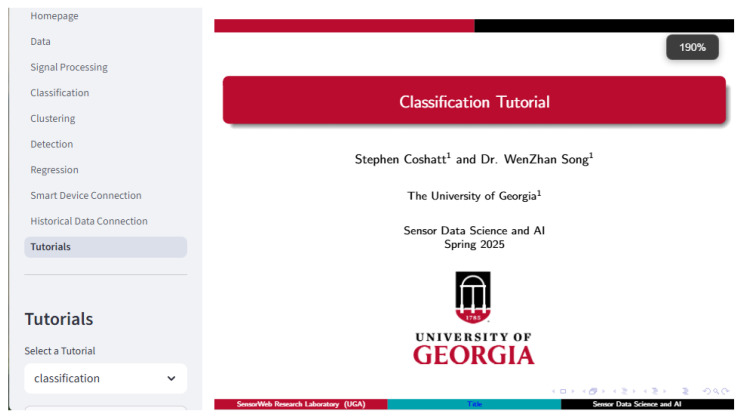
The Tutorial page allows users to view the frameworks associated tutorial slides. Here, the classification tutorial title slide is shown.

The Tutorial page includes menus for the slides that may be viewed on the website and an option to open the Jupyter notebook tutorials in GitHub ^TM^ in a separate browser tab. All tutorial notebooks are set up to open them in Google Colab ^TM^. See Figure 14.

## 3. Results

The framework prototype was first used as a teaching aid in the University of Georgia (UGA) Spring 2024 class, Principles of Cyber-Physical Systems. Using feedback from students and the instructor, the framework design and the tutorials were refined and improved. The framework was used by some of the students to complete homework and project assignments. The framework was used again during the Spring 2025 semester of the same class, to further refine and improve the framework and associated tutorials.

To demonstrate the effectiveness of the framework, a comparison of machine learning models utilized within the framework was performed. Training data was utilized from simulations in previous experiments of the testbed.

### 3.1. Data

The dataset used in this study was generated using a real-world cyber-physical security testbed specifically designed for networked electric drive systems. This testbed, referred to as the CCPS testbed, integrates four electric machine drives—comprising two induction machines and two permanent magnet synchronous machines—each controlled by a TI C2000 TMS320F28335 micro-controller operating under a field-oriented control (FOC) strategy. These drives are embedded within a hybrid IT/OT network environment to emulate realistic control and communication scenarios.

To create high-fidelity cyber-attack data, predefined false data injection attacks (FDIAs) and step-stone attacks through software back doors embedded in the digital signal controllers were created and implemented. The FDIAs target current feedback and speed reference variables, introducing precise distortions in the control loop. These attacks were activated under controlled conditions to maintain system safety while ensuring authentic physical responses. Data reflecting system behavior under normal operation and various attack conditions were collected in real time using NI cDAQ-9132 hardware and streamed to an InfluxDB time-series database.

The sample rate was 10 kHz, which is the maximum sample rate for the ElectricDots. Data was collected at varying motor speeds: 400, 800, 1200, 1300, and 1600 rpms. Data was collected with and without loads (1 V, 2 V, 3 V) at each speed. Labels were also generated along with each case. The FDIA attacks injected a constant into one or two of the three-phase current readings of the motor’s control unit. FDIA type “1” injected a constant into Phase A. FDIA type “2” injected a constant into both Phase A and Phase B. The constant values injected were a percentage of the amplitude added to the actual amplitude and varied as follows: 0.5, 1.0, 2.0, 2.5, 3.0, 3.5, 4, 4.5, and 5 percent.

Sensor readings from the motors, DC bus, and point of common coupling (PCC) were integrated via a dedicated sensor board, enabling the collection of diverse electrical signatures across all scenarios. The dataset was further enriched with synchronized waveform snapshots and labeled attack scenarios, facilitating both machine learning-based fault classification and cyber-attack diagnostics.

The current operating version of the ElectricDots sends out readings to the InfluxDB once every second. Note that the ElectricDots sends the following readings: Voltage, current, the PMU data (amplitude, frequency, and phase angle) of the voltage and current, the power, reactive power, apparent power, the power factor, the total harmonic distortion of the current and voltage, and the temperature of the device. This is conducted so that various features can be used for studies by the CCPS and so that optimal broadcast rates can be determined. In preparation for the demonstration of the framework, the dataset discussed above was downsampled to 1 sample per second to match the current configuration. Only the current from the PCC was used in this demonstration.

### 3.2. Framework Model Comparisons

The data is divided into windows of five samples, with each window containing the raw single-phase current waveform data. There were 63 samples in the original data. After breaking the data into windows, there are 378 cases. There are 78 normal cases, 120 FDIA 1 cases, and 180 FDIA 2 cases.

For the model comparisons, this data was used to test and train a version of each of the models shown in Table 3. Each model was trained and tested ten times with a random test-train split of 0.25. A five-fold cross-validation was used for each run. The Accuracy, weighted Precision, weighted Recall, and weighted F1 Score of each run were recorded; the average is shown in Table 3, and the standard deviation is shown in Table 4.

The best-performing models (score 95% or higher) were tree-based ensembles (bagging, Random Forests, Extra Trees, AdaBoost, Gradient Boost, Histogram Gradient Boost), Decision Tree, and K Nearest Neighbors (and its times series variant). Ensembles are a collection of models. Each model within an ensemble is called an estimator. Bagging models are composed of multiple estimators working in parallel on the same input. Voting is performed on the output of all estimators, which can be weighted or unweighted. Boosting models are simply serialized ensembles. Each estimator makes decisions based on the output of the prior estimator, with the last estimator making the final decisions. The bagged tree models are the bagging, Extra Trees, and Random Forests. The boosted models are AdaBoost, Gradient Boosting, and Histogram Gradient Boosting.

**Figure 15:**
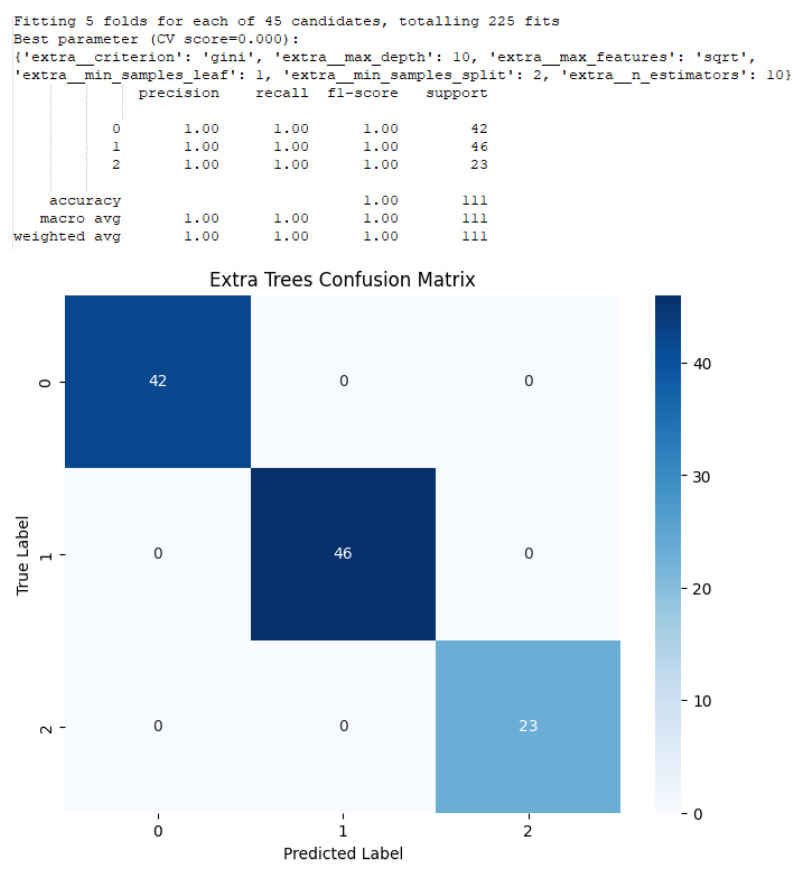
This image is an example of classification output for the results of an Extra Trees model. The parameter settings and the metrics as shown in the text. A confusion matrix is also shown.

For all of the tree-based models, the grid search was performed over a tree maximum depth of 3, 5, 25, and 50. The maximum depth of a decision tree is the number of nodes to pass through before reaching the deepest leaf in the tree. The number of estimators in the ensembles chosen for the grid search was 10, 50, and 100. A sample output for an Extra Trees model is shown in Figure 15.

Nearest neighbor models assume that like things exist in close proximity to each other. The KNN model compares a new input to all previous assigned inputs, and takes the mode of the *k* closest inputs’ assigned labels, close being a distance measure, usually Euclidean distance. The KNN and TS KNN are essentially the same models. However, the TS KNN has a similarity measure called Global Alignment Kernel (GAK) that is specific to time-series data [22]. For these two modes, a grid search for *k* was performed over the following values: 3, 5, and 10.

## 4. Discussion

The primary novelty of the framework is its incorporation of digital signal processing. This feature was distinctly lacking in the other free Python-based frameworks. Secondly, the Sensor AI Framework allows for directly connecting models to live smart devices and databases. Other than H20 AutoML, the Sensor AI Framework utilizes a graphical user interface, and its underlying code can be accessed directly if a user prefers. Python libraries not currently incorporated into the framework can be added. Sktime [23] is a good candidate for later additions, as it focuses on time-series data and uses a Sklearn-type interface. While there are products that could be purchased with more capabilities, the Sensor AI Framework is a well-suited, free, open-source resource for quickly learning and implementing Python machine learning.

The framework was successfully demonstrated using testbed data for model comparison and evaluation. Using the framework, models were created that could correctly classify normal operation and FDIA attacks on the motor operating at different speeds. The best performing models were bagging with decision trees and Random Forests.

Additionally, its tutorial component was successfully used as a teaching tool in a UGA graduate class. Some students used the frameworks for relevant projects. Feedback was positive from both classes in which the framework was used.

However, it is currently limited to machine learning models that have been integrated into the framework. Other frameworks include deep learning, which is lacking in this framework and is thus not suitable for those wishing to use deep learning. The speed at which the framework can train and test models is limited by the computing resources accessible to student research.

## Figures and Tables

**Table 1:** Core modules.

Module File Name	Module Information
classification.py	Classification, Supervised Anomaly Detection
clustering.py	Clustering
detection.py	Unsupervised Anomaly Detection
dsp.py	Digital Signal Processing
regression.py	Regression
utils.py	Plotting and other Miscellaneous functions

All module files are in the “lib” folder in GitHub.

**Table 2:** Tutorials.

Module	
Classification	classification_slides.pdf, classification_tutorial.ipynb
Clustering	unsupervised_slides.pdf, unsupervised_tutorial.ipynb
DSP	dsp_slides.pdf, dsp_tutorial.ipynb
Regression	regression_slides.pdf, regression_tutorial.ipynb
Supervised Detection	classification_slides.pdf, classification_tutorial.ipynb
Unsupervised Detection	unsupervised_slides.pdf, unsupervised_tutorial.ipynb

All tutorial files are in the “tutorial” folder in GitHub.

**Table 3:** The average scores for each model of ten runs are shown in the table below. Each model was scored on Accuracy, Precision, Recall, and the F1 Score. Note that the weighted Accuracy, Precision, and Recall are presented here due to imbalances in the number of each class in the data.

Model Type	Average Scores from 10 Runs			
	Accuracy	Precision	Recall	F1 Score
Bagging	0.9989474	0.9989864	0.9989474	0.9989519
Random Forest	0.9968421	0.9970276	0.9968421	0.9968620
Extra Trees	0.9957895	0.9959658	0.9957895	0.9958041
AdaBoost	0.9936842	0.9937881	0.9936842	0.9936572
Decision Tree	0.9926316	0.9927936	0.9926316	0.9926280
K Nearest Neighbors (KNNs)	0.9905264	0.9908982	0.9905264	0.9904392
Time-Series KNN	0.9905264	0.9908982	0.9905264	0.9904392
Gradient Boost	0.9884211	0.9899116	0.9884210	0.9884201
Histogram Grad. Boost	0.9747369	0.9759421	0.9747369	0.9746843
Non-Myopic Early	0.9347368	0.9418987	0.9347368	0.9350837
Radius NN	0.8663157	0.8777572	0.8663157	0.8675915
Support Vector Classifier (SVC)	0.7389473	0.7742732	0.7389473	0.7320654
Time-Series SVC	0.7031580	0.7500177	0.7031580	0.6967196
Multilayer Perceptron	0.6557896	0.6283973	0.6565679	0.6264954
Linear Discriminant Analysis	0.6147367	0.4935034	0.6147367	0.5459568
Gaussian Naive-Bayes	0.6063158	0.6692445	0.6063158	0.5770321
Quadratic Discriminant Analysis	0.5989473	0.5934619	0.5989473	0.5924451
Gaussian Process	0.5852632	0.5035906	0.5852632	0.5129866
Nearest Centroid	0.5421051	0.6064616	0.5421051	0.5019513
Passive-Aggressive	0.5010525	0.3211840	0.5010525	0.3677192
Bernoulli Naive-Bayes	0.4842105	0.2359225	0.4842105	0.3168296

**Table 4:** The standard deviation for each model of ten runs is shown in the table below. Each model was scored on Accuracy, Precision, Recall, and the F1 Score.

Model Type	Standard Deviation of Scores from 10 Runs			
	**Accuracy**	**Precision**	**Recall**	**F1 Score**
Bagging (trees)	0.0033286	0.0032053	0.0033286	0.0033144
Random Forest	0.0071048	0.0066445	0.0071048	0.0070537
Extra Trees	0.0101694	0.0097522	0.0101694	0.0101381
AdaBoost (trees)	0.0113155	0.0111929	0.0113155	0.0113950
Decision Tree	0.0131754	0.0129107	0.0131754	0.0131879
KNN	0.0195045	0.0186991	0.0195045	0.0198083
Time-Series KNN	0.0195045	0.0186991	0.0195045	0.0198083
Gradient Boost	0.0207285	0.0174075	0.0207286	0.0208220
Histogram Grad. Boost	0.0281578	0.0265375	0.0281578	0.0282982
Non-Myopic Early	0.0429161	0.0366547	0.0429161	0.0423254
Radius NN	0.0732739	0.0661957	0.0732739	0.0717121
SVC	0.0396352	0.0312425	0.0396352	0.0435080
Time-Series SVC	0.0640867	0.0721340	0.0640867	0.0626575
Multilayer Perceptron	0.0543689	0.1234110	0.0538698	0.0796094
Linear Discriminant Analysis	0.0358514	0.0298841	0.0358514	0.0334393
Gaussian Naive-Bayes	0.0484160	0.0671592	0.0484160	0.0472427
Quadratic Discriminant Analysis	0.0395264	0.0389216	0.0395264	0.0387197
Gaussian Process	0.0394486	0.0481147	0.0394486	0.0474366
Nearest Centroid	0.0228744	0.0277980	0.0228744	0.0323248
Passive-Aggressive	0.1406311	0.2279550	0.1406311	0.1838261
Bernoulli Naive-Bayes	0.0403126	0.0394275	0.0403126	0.0441936

## Data Availability

The original data presented in the study are openly available Sensor-WebEdu/SensorAI at https://github.com/SensorWebEdu/SensorAI.git (accessed on 27 September 2025).

## Abbreviations

The following abbreviations are used in this manuscript:

CCPS	Center for Cyber-Physical Systems
CWT	Continuous Wavelet Transform
DBSCAN	Density-Based Spatial Clustering of Applications with Noise
DSP	Digital Signal Processing
ECU	Electronic Control Unit
eDot	ElectricDot
Elastic-Net	Elastic-Net Regularization
EMD	Empirical Mode Decomposition
FDIA	False Data Injection
FFT	Fast-Fourier Transform
FOC	Field-Orient Control
GAK	Global Alignment Kernel
GPU	Graphics Processing Unit
GUI	Graphical User Interface
IT	Information Technology
KNN	K Nearest Neighbor
LARS	Least Angle Regression Shrinkage
LASSO	Least Absolute Shrinkage and Selection Operator
MQTT	Message Queuing Telemetry Transport
OPTICS	Ordering Points To Identify the Clustering Structure
OT	Operational Technology
PCC	Point of Common Coupling
PCT	Polynomial Chirplet Transform
RANSAC	Random Sample Consensus
SCG	Seismocardiography
SSA	Singular Spectrum Analysis
SST	SynchroSqueezing Transform
STFT	Short Time Fourier Transform
THD	Total Harmonic Distortion
TS KNN	Time-Series K Nearest Neighbor
UGA	University of Georgia
WVD	Wigner–Ville Distribution
